# Identifying fungal-host associations in an amphibian host system

**DOI:** 10.1371/journal.pone.0256328

**Published:** 2021-08-19

**Authors:** Alexandra Alexiev, Melissa Y. Chen, Valerie J. McKenzie

**Affiliations:** Department of Ecology and Evolutionary Biology, University of Colorado Boulder, Boulder, Colorado, United States of America; Universidad Nacional Autonoma de Mexico, MEXICO

## Abstract

Host-associated microbes can interact with macro-organisms in a number of ways that affect host health. Few studies of host-associated microbiomes, however, focus on fungi. In addition, it is difficult to discern whether a fungal organism found in or on an ectotherm host is associating with it in a durable, symbiotic interaction versus a transient one, and to what extent the habitat and host share microbes. We seek to identify these host-microbe interactions on an amphibian, the Colorado boreal toad (*Anaxyrus boreas boreas*). We sequenced the ITS1 region of the fungal community on the skin of wild toads (n = 124) from four sites in the Colorado Rocky Mountains, across its physiologically dynamic developmental life stages. We also sampled the common habitats used by boreal toads: water from their natal wetland and aquatic pond sediment. We then examined diversity patterns within different life stages, between host and habitat, and identified fungal taxa that could be putatively host-associated with toads by using an indicator species analysis on toad versus environmental samples. Host and habitat were strikingly similar, with the exception of toad eggs. Post-hatching toad life stages were distinct in their various fungal diversity measures. We identified eight fungal taxa that were significantly associated with eggs, but no other fungal taxa were associated with other toad life stages compared with their environmental habitat. This suggests that although pre- and post-metamorphic toad life stages differ from each other, the habitat and host fungal communities are so similar that identifying obligate host symbionts is difficult with the techniques used here. This approach does, however, leverage sequence data from host and habitat samples to predict which microbial taxa are host-associated versus transient microbes, thereby condensing a large set of sequence data into a smaller list of potential targets for further consideration.

## Introduction

Host-associated microbes provide a number of benefits to hosts that can affect their health [1]. Common examples include microbial symbionts providing nutrients or modulating immune function in the host. Such relationships can sometimes be co-evolved if microbes obtain resources or niche space in return [1–4]. Most studies of host-associated microbes focus on bacteria, as they are likely the most abundant microbial group in and on hosts, but other microbial domains of life are increasingly studied for their roles in host health. Fungi, for example, have often been investigated as pathogenic organisms in relation to hosts but can also be non-pathogenic [5–9]. One such well-established interaction involves Attine ants and the fungus that they grow and maintain as food. These ants selectively remove other non-symbiotic fungi and foster beneficial bacteria that protect the cultivar from contamination [10–13]. Fungi are also particularly important to ectotherms (e.g., fishes, amphibians, reptiles, and invertebrates), in which fungal pathogens are more plentiful due to their body temperature reflecting the outer environment [14]. This phenomenon has also been associated with ectotherm’s microbial community stability in relation to changing temperatures [15–19]. Such research illustrates that fungi can be important components of host-associated microbial communities, yet most remain understudied. The amphibian system described in this study, for example, has been extensively characterized in terms of its bacterial skin inhabitants, but the fungal realm remains largely undiscovered.

**Fig 1:**
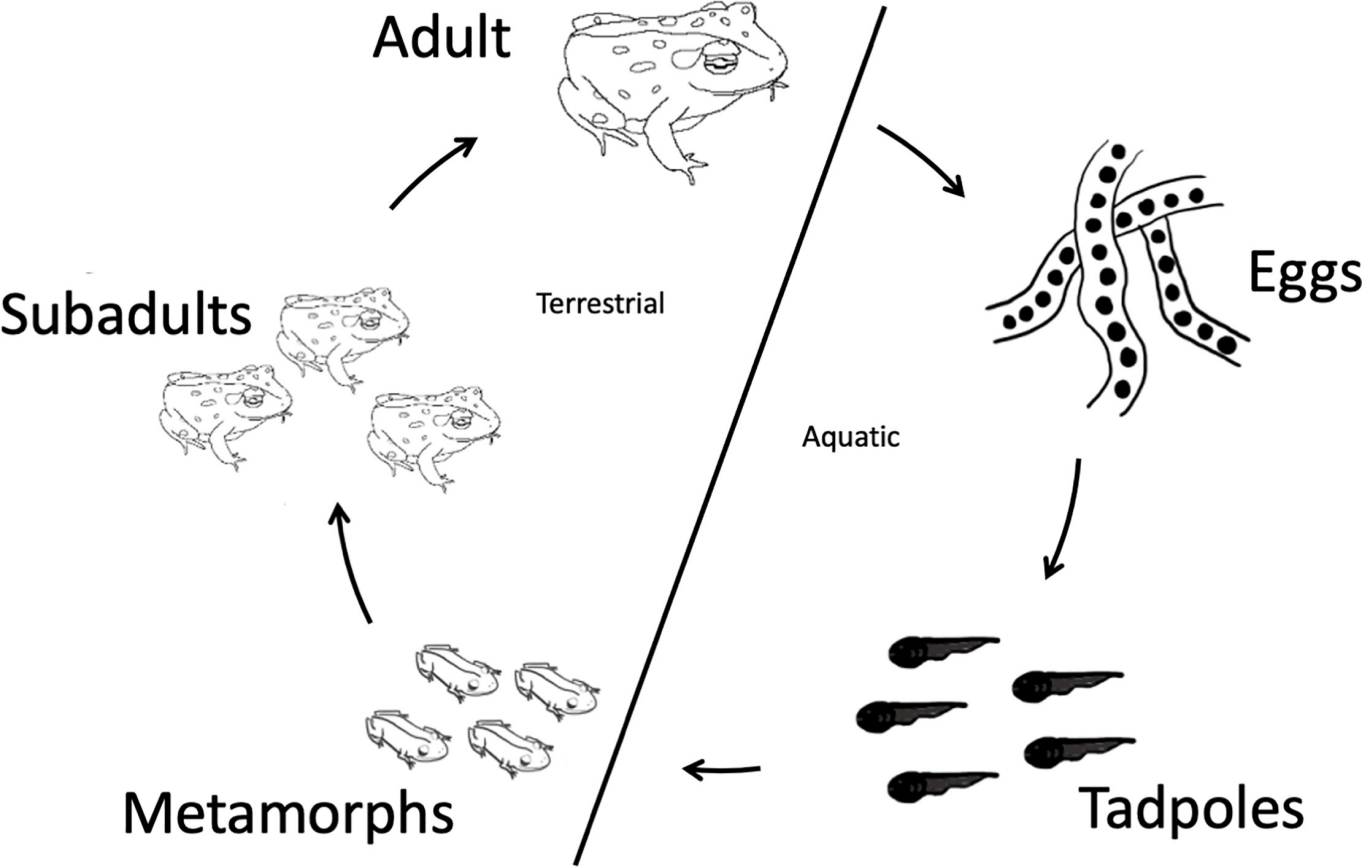
An illustration of the physiologically dynamic metamorphosis that boreal toads go through. The boreal toad life cycle has been linked to changes in their skin bacterial communities. Adults lay eggs in an aquatic habitat, which then hatch into tadpoles. Tadpoles develop into metamorphs and move out of the water, into a largely terrestrial habitat, although they continue to return to the water throughout their lifespan. Metamorphs grow into subadults, which are not reproductively mature for 2–3 years. Once they are reproductively mature, the adults visit pond areas to mate and reproduce during spring and retreat to upland terrestrial habitats to overwinter in underground hibernacula.

Given this knowledge gap, this study explores the fungal community changes as the amphibian host goes through a dynamic metamorphosis, in which the skin and environment interface in ways that have previously been observed in bacterial communities but are little-studied in fungi. The Colorado boreal toad (*Anaxyrus boreas boreas*), is a long-lived animal (up to 20+ years) and inhabits high-elevation wetland habitats in the Rocky Mountains. This organism is a tractable wild host system in which to study microbial community dynamics since it has a thoroughly characterized bacterial community across its various developmental life stages [20–22]. Like many amphibians, toads go through a physiologically dynamic metamorphosis in which their environment (i.e., aquatic or terrestrial) has a significant impact on their microbial communities [20,22–24] (Fig 1). Since the toad’s life cycle spans both aquatic and terrestrial habitats, recent work has characterized the microbial groups on boreal toad skin across these life stages. One study found that fungi dominated boreal toad skin in later life stages but were rare on earlier life stages, which the authors hypothesized was linked to a higher proportion of antifungal-producing bacteria on earlier life stages [24]. Amphibian skin is a mucosal tissue that can harbor thousands of bacterial taxa [20,23,25–27] and, as was recently discovered, fungal taxa as well [24,28,29]. Prest, et al. (2019) investigated bacterial community assembly across the life stages of the boreal toad using the same samples used in this study, and confirmed that boreal toad skin bacterial communities change across development, going through cycles of succession after egg-hatching and metamorphosis [22]. Given this descriptive work, we recognized that it is difficult to establish interactions between hosts and symbionts without knowing which microbes are host-associated versus transient.

This challenge is common to many microbial taxa but is especially true of fungal taxa because many free-living fungi disperse easily via wind-blown spores and can be very widespread throughout the environment [30,31]. Microbial samples collected from wild animals in their natural habitats may, therefore, include a mix of host-associated as well as transient microbes picked up from the environment. Transient microbes, in the context of this paper, refer to microbes that are only temporarily found in association with a host, but they do not persist or grow there [32,33]. For example, transient microbes may come in contact with host animal skin from the environment or may end up in the gut alongside the food that an animal has eaten. Given that many fungi are wind-dispersed and wide-ranging, it can be difficult to discern whether a fungal organism detected in or on a host is associating with that host in a durable, symbiotic interaction [31,34] versus a transient one, particularly with often-used culture-independent techniques.

Disentangling the role that different microbes play in or on a host animal is difficult and ultimately requires careful experimental study of the relationships between hosts and microbes. For many animal systems, however, it is simply not practical to use an experimental, culture-based approach as a first step because there are so many microbes found in association with animals. As mentioned earlier, microbes recovered via sequencing may be prone to having a high number of transient taxa because of their easily dispersed spores and widespread distributions throughout the environment [30,31,35,36]. Thus, we identified indicator taxa from microbial sequence data detected from animal hosts to determine which specific OTUs differed in abundance between host and habitat samples. These results, in turn, could inform follow-up experimental studies that target specific microbes and their possible interactions with amphibian hosts.

This study therefore aims to characterize the fungal community, previously unstudied in this host, on wild boreal toads across its life stages and predict putative host-associated taxa for further experimental study. We asked: (1) how does the fungal community vary across the different life stages of the host, and (2) which fungal OTUs, if any, are significantly associated with individual toad life stages versus environmental habitats. This work distills a large microbial community dataset into a smaller and more manageable set of targets for future culture-based studies, as part of a larger workflow commonly used in the amphibian microbiome community to identify symbionts related to host health [37–39].

## Materials and methods

### Field sampling

We sampled the skin microbial community of the Colorado boreal toad (*Anaxyrus boreas boreas*) in the Rocky Mountains in Chaffee County during the summer of 2015 using an approved IACUC protocol from the University of Colorado (protocol number 1505.04) and a Scientific Collection License from Colorado Parks and Wildlife (license number 15HP99). Samples originated from four sites with active breeding (presence of egg masses or tadpoles), encompassing 10 Gosner life stages in the boreal toad [40]. These were categorized into the following Gosner stages: eggs; tadpoles [20–35,39,40]; metamorphs; subadults; and adults. “Eggs” in this study refers to developing embryos that have been laid by the female in the environment, fertilized by a male, and develop over time, resting on the sediment in the water. In addition, we collected water and aquatic sediment samples from the sites, as available. The sites are all within a 30-mile radius of each other, each along an independent creek within the same watershed. They ranged in elevation from about 9,500 feet at Four Mile to 11,000 feet at Denny’s Creek. Three of the sites (Denny’s Creek, South Cottonwood, and Collegiate Peaks) are located in the Collegiate Peaks Wilderness area, and one site (Four Mile) is located in the Buffalo Peaks Wilderness area. These are wetland sites with slow-moving, marshy beaver ponds alongside snow-melt fed streams. We visited sites up to four times each between May and September. Since this was a field sampling design that relied upon the availability of animals and access to remote mountain field sites, our experiments have unbalanced sample sizes, but we aimed to have enough replicates that our analyses would be informative (Table 1). The environmental samples represent the substrates that toads at the sites were observed interacting with (i.e., water and sediment at the specific place where egg and tadpole swabs were collected). Metamorphs were collected from water, where they spend much of their time, although they are also terrestrial. Subadult and adult toads hide in burrows and winter hibernacula underground where they contact soil intimately (both in length of time and surface area of contact), in addition to plant matter. We were unable to sample these areas since this would disturb or destruct critical habitat for this endangered species. There are infinite environmental habitats and microhabitats in the alpine wetland, and we sampled the most immediately observable areas that toads had direct contact with that were also accessible.

**Table 1:** Sample numbers per each sample type from four locations.

	*4 Mile*	*Collegiate Peaks*	*Denny’s Creek*	*South Cottonwood*	Total
*Sediment*	6	2	7	10	25
*Water*	3	0	7	10	20
*Eggs*	8	1	3	0	12
*Tadpole*	16	0	29	21	66
*Metamorph*	2	0	0	23	25
*Subadult*	2	3	0	0	5
*Adult*	5	0	10	1	16
*Total*	42	6	56	65	169

Samples that passed DNA extraction, sequencing, and quality filtering and were therefore used in fungal community and indicator OTU analysis.

We collected boreal toad skin samples with sterile rayon swabs (BBL culture swabs from BD^TM^), following methods described previously [25]. The outer coat of egg masses, pond water, and pond sediment were sampled using sterile rayon swabs as described in Prest et al. (2018) [22]. The egg samples we took represent the microbes on the outer surface of the jelly coat and may not reflect any microbes (if present) directly on the embryos within it. Animals were returned to the site of capture post-sampling. We recorded Gosner stage for each individual sampled using a hand-held lens to determine the developmental stage within a range of two Gosner stages (ex. the earliest stage post-hatching is estimated to be between Gosner stages 20–22). It is likely that within each wetland, the tadpoles were siblings that emerged from a single egg mass at each respective site, since only one egg mass was observed at each of the four sites. Table 1 shows only samples that passed DNA extraction, sequencing, and quality filtering for use in downstream analyses, which resulted in 169 samples collected across four different sites in CO, encompassing two types of environmental samples and 10 different life stages in boreal toad.

### Sample processing

We extracted DNA from swabs using the MoBio PowerSoil Extraction kit (MoBio Laboratories, Carlsbad, CA, USA) using the standard protocol procedure with the following modifications for low biomass material [41]: samples were incubated for 10 min at 65°C after the addition of C1; time spent vortexing with powerbeads was increased by 5 min; the C6 solution was added to the spin column filter and allowed to incubate at room temperature for 5 min before the final elution. PCR amplification used primers ITS 1f-ITS 2 (10μM concentration) to target the ITS1 gene region (EMP.ITSkabir from the Earth Microbiome Project), as these would detect a broad diversity of fungal taxa, have well-curated reference databases, and are used by other amphibian researchers, allowing for future comparison between multiple amphibian species. We combined 12.5 μL of GoTaq G2 colorless master mix (Promega, Madison, WI, USA), 10.5 μL of sterile water, 0.5 μL of each primer, and 1 μL of DNA template into each reaction. The thermocycler settings were as follows: 94°C for 3 min, then 35 cycles of 94°C for 45 s, 50°C for 60 s, and 72°C for 90 s, and a final extension at 72°C for 10 min. PCR was performed in triplicate in plates to obtain sufficient material for sequencing. One of each sample triplicate was checked on a 2% agarose gel for successful amplification. Triplicates were then combined and quantified via a Quant-IT PicoGreen dsDNA assay kit (Thermo Fisher Scientific, Waltham, MA, USA) and pooled using equimolar amounts of PCR amplicons. Pooled DNA was cleaned with a MoBio UltraClean PCR cleanup and DNA purification kit (MoBio Laboratories, Carlsbad, CA, USA) and re-quantified using the PicoGreen reagent as before. Equimolar amplicons were subsequently pooled and quantified for final DNA concentration using a Qubit 3 Fluorometer (Thermo Fisher Scientific, Waltham, MA, USA).

During the sample processing, we experienced difficulty obtaining PCR product from samples collected from early-stage tadpoles and eggs. This is consistent with previous research, which found that both the number of fungal OTUs as well as fungal DNA (as a proxy for fungal biomass) are lowest on early developmental life stages [24]. As a result, we successfully sequenced a smaller portion of the tadpole and egg samples than what was collected, relative to other sample types. S1 Fig shows which sample types filtered out at different sample processing steps from sample collection to analysis. We still managed to recover fungal DNA from a sufficient number of tadpole and egg samples (n = 66 and 12, respectively; Table 1).

### DNA sequencing, quality filtering, and fungal OTU assignment

Samples were sequenced on an Illumina MiSeq sequencer (Illumina, San Diego, CA, USA) at the Biofrontiers Next-Generation Sequencing Facility at University of Colorado, Boulder. Sequencing yielded 9,357,574 total sequences for 205 samples on the run, which included two different kinds of negative controls. These comprised (1) DNA extraction kit controls, which had no input DNA but underwent the entire extraction process alongside all the other samples, and (2) PCR negative controls, which were PCR reactions with no template DNA. We then used Decontam version 1.1.2 [42], an R package that uses the frequencies or prevalences of OTUs across samples compared to controls, to identify likely contaminants there were then removed from all samples. We used both the frequency and prevalence method in Decontam to identify potential contaminants and, as the OTUs identified were mostly overlapping between the methods with some differences, we removed all OTUs identified by either method. See S1 Table for the taxonomy of these OTUs.

We assigned fungal OTUs at 97% and 99% identity and matched the consensus sequence to the UNITE database version 7.2 2017-12-01 [43] using USEARCH v10.0.240_i86linux64 [44]. USEARCH filtered chimeras in the process of assigning OTUs. We used MacQIIME 1.9.1–2015 [45] to filter chloroplasts, mitochondria, and singletons from the dataset as well. This approach was based on the following workflow: https://github.com/amoliverio/data_processing/blob/master/data_processing_tutorial.md. Exploratory diversity analyses were largely consistent between 97% and 99% identity clustering, but we chose 97% since some of the fungal taxa we identified had large genetic variability within the same phylogenetic group and were clustered into “unidentified” groups at 99% identity.

### Mycobiome variation between life stages and environmental sample types

We measured alpha and beta diversity using the R packages mctoolsr (version 0.1.1.2) [46] and vegan (version 2.5–6) [47]. We used ggplot2 (version 3.2.1) [48] to visualize diversity patterns. Data was rarefied to 1,030 sequences per sample. During initial data exploration, we used a pairwise Mann-Whitney test with Bonferroni correction on OTU species richness and a pairwise PERMANOVA on Bray-Curtis dissimilarity to determine if tadpoles at different Gosner stages had similar enough fungal communities to be combined into one sample group for further diversity analyses. Tadpoles were not statistically different in either richness or composition, so we combined them in subsequent analyses. To characterize fungal alpha diversity, we used a Kruskal-Wallis test with a Bonferroni correction to evaluate the statistical significance of three alpha diversity measures (OTU richness, Simpson’s metric, and Shannon index), which are in the supplement (S2 Fig).

We calculated three beta diversity measures (Bray-Curtis, weighted and unweighted UniFrac) since each metric measures a slightly different aspect of community dissimilarity. Bray-Curtis and weighted UniFrac use relative abundance to calculate beta diversity (as opposed to prevalence used in unweighted UniFrac), and both types of UniFrac incorporate additional phylogenetic information in the beta diversity measure (whereas Bray-Curtis does not). We used a Mantel test to see if the data frames were correlated, thus assessing whether all three beta diversity matrices capitulated the same patterns. The beta diversity data frames were correlated (Partial Mantel test using Spearman Rank Coefficients; R = 0.3229, p = 0.001, permutations = 999), so we chose the Bray-Curtis metric in the main figures and interpretations. We used a pairwise PERMANOVA and PERMDISP with a Tukey’s HSD test to evaluate beta diversity (Bray-Curtis dissimilarity). We were primarily interested in community differences among sample types, but site had a small, significant effect on community dissimilarity. Sites were within the same watershed in the same county, so although they might be different on a very local scale, they are ecologically similar on a regional scale. Thus, site was used as a ‘strata’ in the PERMANOVA script when testing whether the fungal microbial community varied across sample type. Further, to ensure that each site had comparable patterns, we performed alpha and beta diversity analyses on each site individually and patterns were visually consistent with each other, but there were too few samples to adequately perform statistical tests.

### Identifying host-associated fungal taxa

We used an indicator species analysis called ANCOM to identify the fungi associated with a sample type, in our case toad (across each individual life stage) or environmental samples. ANCOM uses a log ratio transformation of relative abundance data to ask whether relative bacterial species ranks differ between two sample types. This approach reduces intra-sample correlations between species proportions in compositional data. We found that ANCOM was fairly conservative in its estimates considering the high possibility of false positives in other existing indicator species methods derived from microbial sequence data, as was expected given previous tests comparing its rigor to other methods [49]. We adapted the original ANCOM R code (https://github.com/mka2136/lt_microbiome/blob/master/inputs/ANCOM_updated_code.R) to include effect sizes of each OTU [50]. The updated code is found at (https://github.com/mech3132/MSC_code/blob/master/ANCOM_updated_code_MYC_AA.R). The graphs for this portion of the analysis were created using ggplot2 [48].

We interpreted fungal OTUs that were significantly abundant in toad samples as putatively host-associated based on ANCOM’s modeling of the sequence data, and only made biological interpretations based on genus or higher phylogenetic resolution since the sequencing methods we used do not allow for confidence at the species level, which would require longer read sequencing. It is also difficult to know for sure whether low sequence presence reflects a low abundance living microbe (at the time of sampling) or relic DNA. To account for this phenomenon, we filtered out OTUs with only one instance and constrained results to OTUs that were well-represented in the data (i.e., were present in multiple samples within each sample type).

We ran ANCOM to identify indicator taxa in toad versus environmental samples and looked for those that had a high W statistic (how many times the model recovered that OTU as being significant) and high Centered Log Ratio (CLR) test F statistic (effect size). Since the fungal community analysis revealed that life stages and habitat types are significantly different, we ran ANCOM in a pairwise fashion between each toad life stage and each habitat type (e.g., water-adult, sediment-tadpole, etc.). The W stat cut-off is chosen by the ANCOM model using the cumulative probability distribution of the W stat value (S3 Fig). Only sediment-egg and water-egg comparisons had significantly varying fungal OTU’s. For each of these, we graphed the relative abundances in toad versus environmental samples for each OTU, as an intuitive way to corroborate the ANCOM results. Taxa with a significant W stat, high effect size, and higher relative abundance in toad samples are “host-associated” and taxa with a significant W stat, high effect size, and higher relative abundance in the environment are “transient.” For each of these taxa, we compared the exact ITS sequence to other reports of that sequence in the UNITE database to determine whether it made biological sense for these taxa to be on boreal toad skin and to confirm how close each sequence match was to the reference sequence used to assign taxonomy.

## Results

### How do fungal OTUs vary between life stages and environmental sample types?

After merging, quality filtering, and rarefying, there were 168,627 sequences representing 2,156 fungal OTUs across 169 samples. This data encompassed five life stages and two environmental sample types across four different site locations (Table 1).

Fungal diversity was different across life stages and between toads and their environment, but only to a small degree, and there was overlap in many of the taxa across host and habitat. Overall, fungal diversity patterns recapitulate bacterial community patterns found in previous research in terms of dissimilarity and dispersion between microbial communities across different life stages and environmental samples [22].

We also evaluated our sampling scheme and whether we should put certain sample types, like tadpoles, together. Despite unbalanced sampling, fungal communities from the least-sampled sample type (subadult, n = 5) had high similarity. PERMANOVA and PERMDISP account for unbalanced sampling schemes when calculating significance between group centroids and dispersions in the ordination [51,52]. A rarefaction curve of the data before rarefying shows that the curves for each sample type reach saturation (S4 Fig). All alpha and beta diversity measures of the tadpole mycobiome were similar between Gosner stage intervals, so they were pooled into one “tadpole” sample group for hypothesis testing (S2 Table).

**Fig 2:**
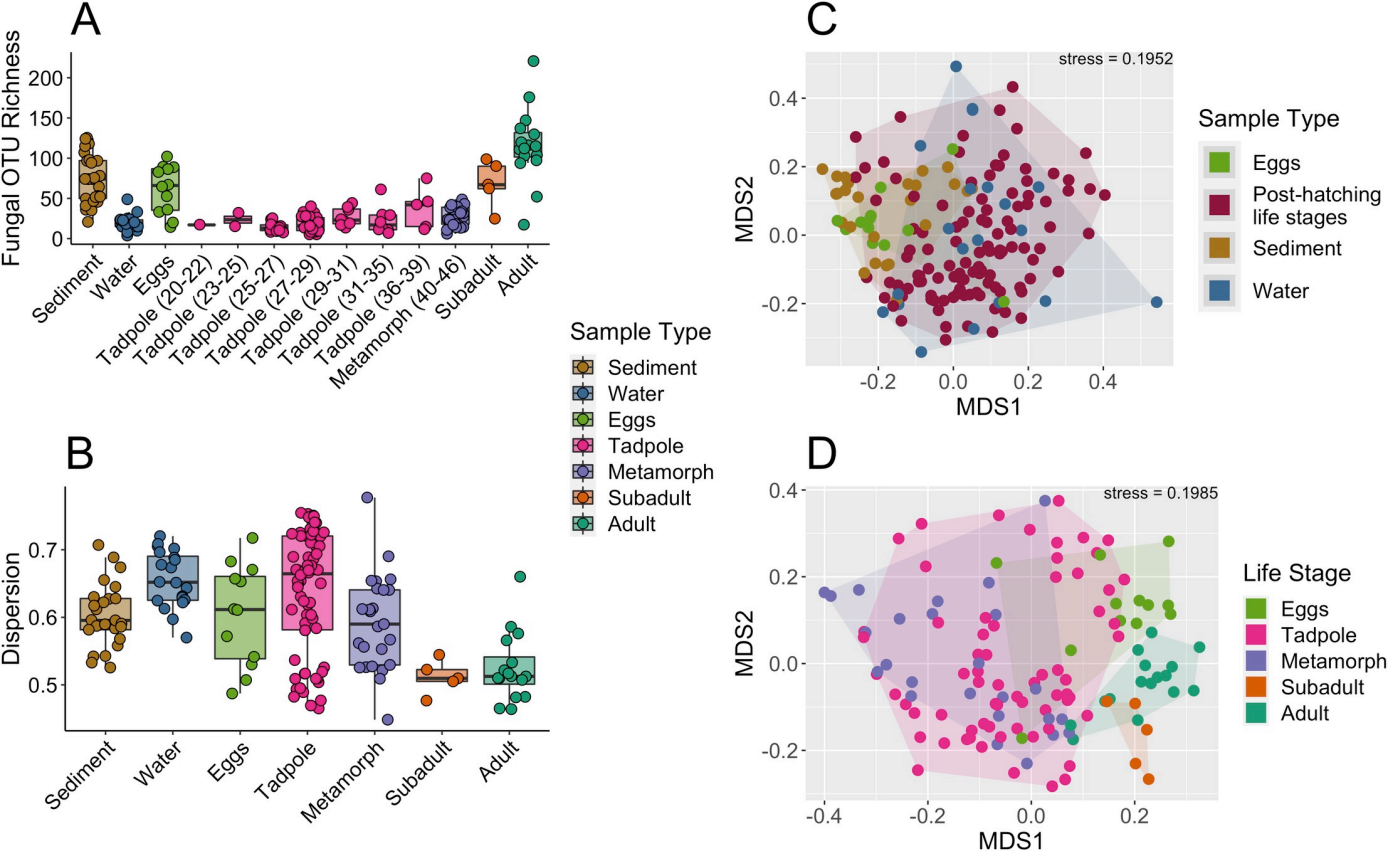
Alpha and beta diversity of fungal communities on boreal toads and environmental samples (n = 169). (A) Fungal OTU richness among different sample types (Kruskal-Wallis with Bonferroni p-value adjustment, KW chi-squared stat = 102.79, p = 2.05e-15). The numbers after the younger developmental stages indicate the Gosner stage range. (B) Boxplot showing the dispersion of points per sample type based on the ordination analysis (see PERMDISP results in S3 Table). (C) NMDS plot showing the Bray-Curtis dissimilarity among all the samples, color represents the different sample types of toad (eggs and post-hatching life stages) and environment (sediment and water) (PERMANOVA, R^2^ = 0.06192, p = 0.001). (D) NMDS plot showing the Bray-Curtis dissimilarity among the toad samples, where color represents the different life stages (PERMANOVA, R^2^ = 0.08723, p = 0.001).

OTU richness and Shannon diversity were different across life stages and environmental samples (Kruskal-Wallis with Bonferroni p-value adjustment; p = 2.9e-15 and p = 6.1e-06, respectively) (Fig 2A). While eggs, subadults, and adults were comparable in richness to sediment, tadpoles and metamorphs had a similarly low richness to water (Fig 2A).

We used PERMANOVA to assess whether beta diversity centroids were significantly different among the sample types, and PERMDISP to assess whether the dispersion of the points in each sample type was significantly different. The life stages and different environmental sample types were significantly different (PERMANOVA, R^2^ = 0.1079, p = 0.001; see S4 Table for pairwise PERMANOVA statistical results), and tadpoles and metamorphs were differentiated from the other life stages by higher dispersion of fungal beta diversity (Fig 2B, S3 Table shows the PERMDISP statistical results). An alternate version of the dispersion figure shows that tadpoles of early and late stages vary across the entire range shown in the boxplot in Fig 2B, and therefore there is no skew in different tadpole stages having specific dispersions (S5 Fig). On the NMDS plot (Fig 2D), subadults and adults had some of the lowest dispersions of their points, whereas tadpoles had the most dispersed communities (Fig 2B and 2D, S3 and S4 Tables for statistics).

### Which fungal OTUs are significantly associated with toads versus the environment?

**Fig 3:**
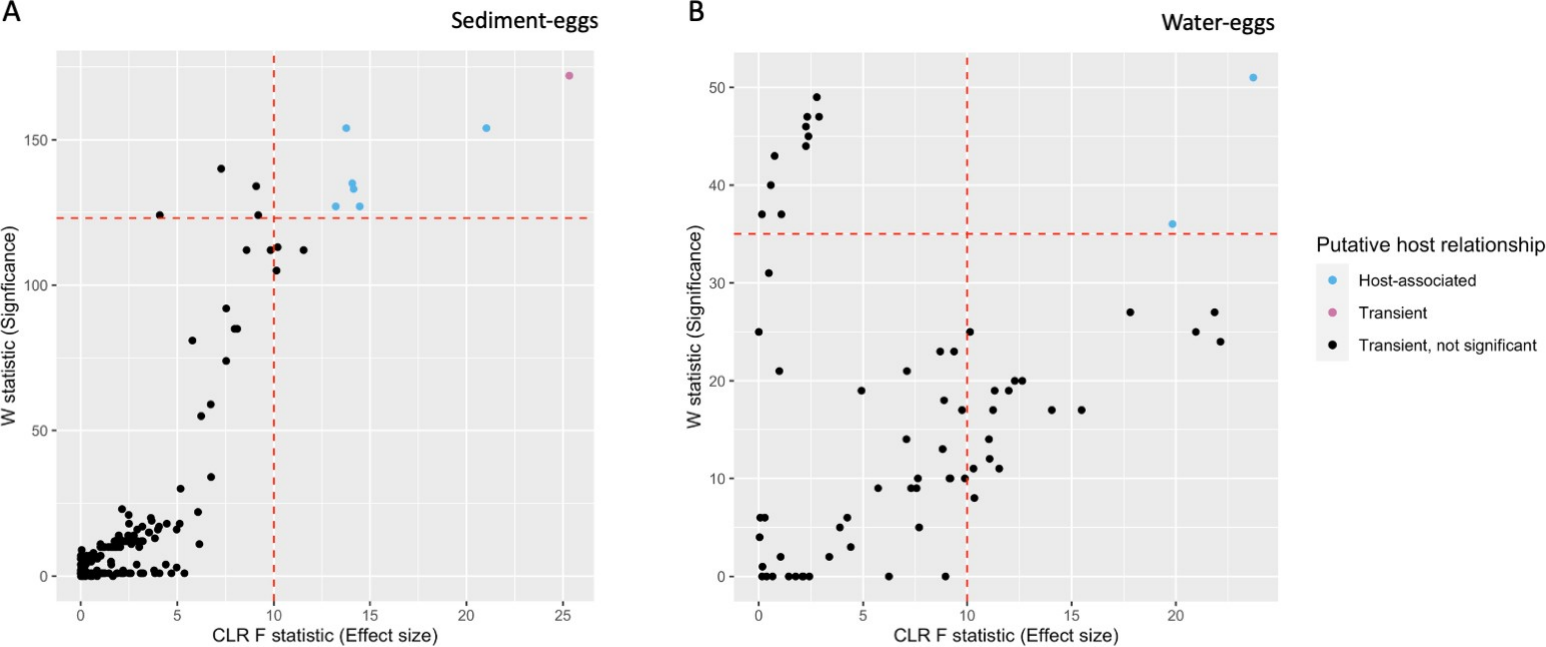
A volcano plot showing the ANCOM model W statistic (how many times the model recovered that OTU as being significant) and Centered Log Ratio (CLR) test F statistic (effect size). (A) Shows the sediment-egg OTUs, whereas (B) shows water-egg OTUs. The horizontal red dotted line in each graph shows the W statistic cut-off (above the line, i.e., high W statistic) between the group of fungal OTUs we identified as being significantly associated with either the host samples (potential host-associated taxxa) or the environmental samples (potential transient taxa). The vertical red dotted line in each graph represents the effect size cut-off the ANCOM model chose as most conservative. Each dot is an OTU and is colored by the putatively host-associated (blue), and putatively transient (purple). OTUs that were not significant and had low area effect size are black, and we interpreted these as fungi that are transient but not significantly enriched in the environment relative to toad hosts. The volcano plot shows that this group of OTUs is distinct in its W statistic values compared to other OTUs (i.e., their association with a toad or environment sample type is stronger than most other OTUs in the dataset).

**Fig 4:**
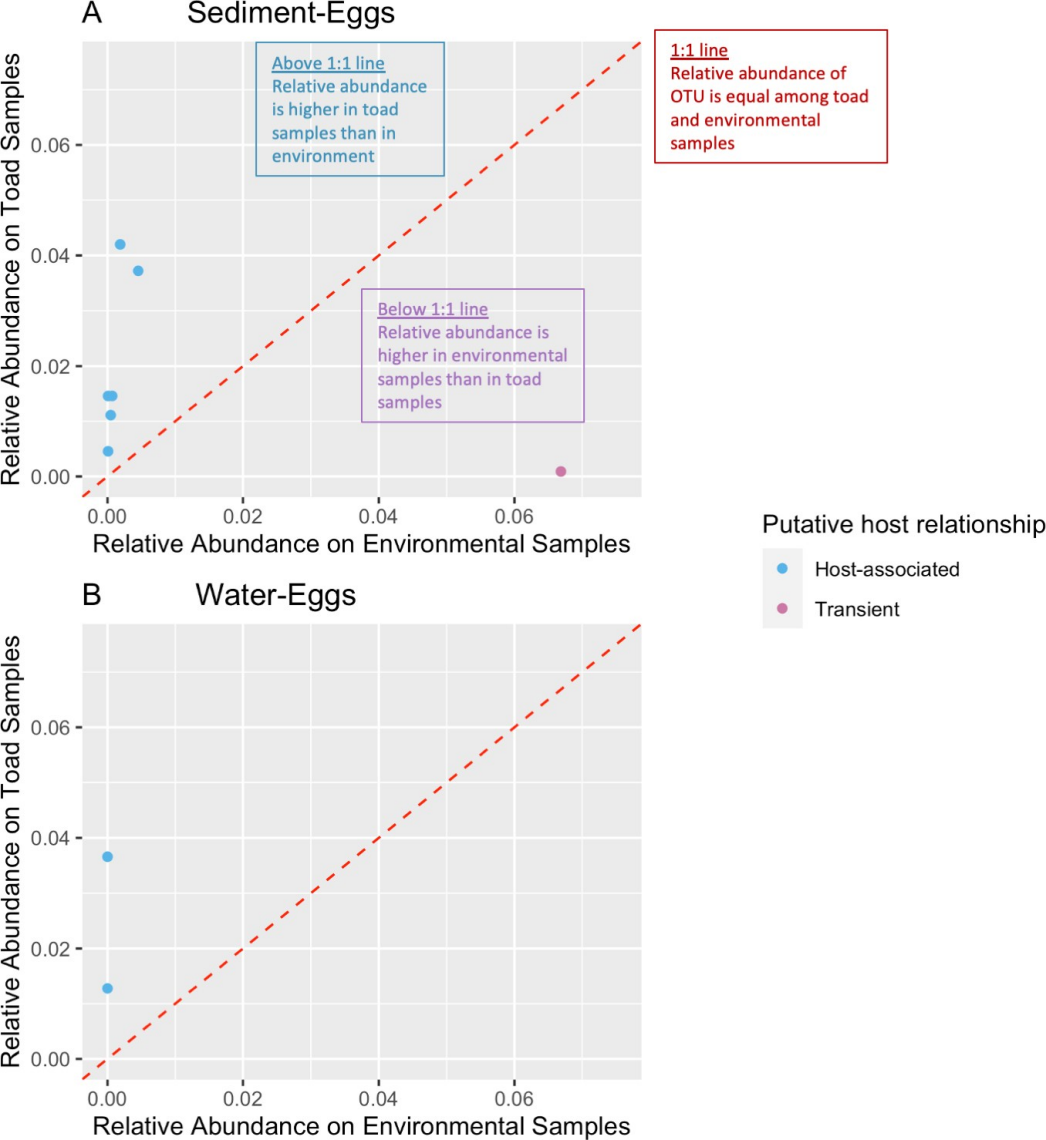
Significant indicator fungal taxa graphed by their relative abundance in toad versus environmental samples. (A) Shows the sediment-egg OTUs, whereas (B) shows water-egg OTUs. The colors denote putatively host-associated (blue) versus putatively transient (purple) fungal OTUs, whose UNITE taxonomy is listed in Table 2. The categorization of “host-associated” or “transient” was attributed using the ANCOM analysis results (i.e., taxa with a significant W stat, high effect size, and higher relative abundance in toad samples are “host-associated” and taxa with a significant W stat, high effect size, and higher relative abundance in the environment are “transient”). Here we have graphed the relative abundance in toad samples compared to environmental samples for each of these significant OTUs for interpretation purposes.

ANCOM identified nine fungal OTUs that were significantly enriched when comparing sediment-egg and water-egg (all other comparisons yielded no significant results), eight of which were more highly abundant in egg samples (Figs 3 and 4, Table 2). Since ANCOM uses rank abundance to determine indicators of toad or environmental samples, we plotted the OTUs’ relative abundance in toads versus relative abundance in the environment to visualize the significant fungal OTUs as putatively host-associated versus transient (Fig 4). The 1:1 line in Fig 4 represents OTUs whose relative abundance on toads is the same as in the environment, and are therefore putatively transient. Anything below the 1:1 line is also putatively transient as it is more abundant in the environment than on toads. OTUs falling above the 1:1 line are putative host-associated fungal taxa as they are in a top relative abundance in toad samples but bottom relative abundance in the environmental samples. Table 2 supports these results, listing the nine significant fungal OTUs (putatively host-associated and transient) and their relative abundance as well as the statistics from ANCOM.

**Table 2:** Putative host-associated (n = 8) and transient (n = 1) fungal OTUs identified in this data using ANCOM.

*OTU Taxonomy*	*OTU_ID*	*Putative Niche*	*Comparison*	*W statistic*	*CLR F statistic*	*relative abundance in environment*	*relative abundance on toad*
*g__Dactylaria*	OTU_36	Host-associated	Water-Egg	51	23.72	0.0000000	0.0365782
*g__Phenolifera*	OTU_62	Host-associated	Water-Egg	36	19.84	0.0000000	0.0127718
*g__Cladosporium*	OTU_4	Host-associated	Sediment-Egg	154	21.03	0.0018676	0.0419865
*g__Sclerostagonospora*	OTU_12	Host-associated	Sediment-Egg	154	13.76	0.0045347	0.0372073
*g__Phaeosphaeria*	OTU_6083	Host-associated	Sediment-Egg	135	14.08	0.0000861	0.0145974
*g__Volucrispora*	OTU_106	Host-associated	Sediment-Egg	133	14.15	0.0006875	0.0146095
*g__Rhynchosporium*	OTU_181	Host-associated	Sediment-Egg	127	14.47	0.0004722	0.0111088
*o__Helotiales*	OTU_430	Host-associated	Sediment-Egg	127	13.22	0.0000861	0.0045531
*p__Rozellomycota*	OTU_34	Transient	Sediment-Egg	172	25.33	0.0668613	0.0009058

The W statistic and CLR F statistic were used to determine the significance and effect size of fungal OTUs that were indicators of toad versus environmental samples. The relative abundance is shown in the table, and these trends are corroborated by ANCOM (which compare relative rank changes between OTUs to identify indicator species). The letters preceding the taxa names indicate what phylogenetic grouping each taxon name corresponds to (i.e., “g__” is for genus, “o__” is for order, “p__” is for phylum).

One of the fungi recovered as a “host-associated taxa” was a group containing known amphibian pathogens (*Cladosporium*) [53,54], and animals appeared healthy when sampled. 37 sequences in the dataset were identified in the phylum Chytridiomycota with the UNITE reference database, but these were at most 139 sequences per sample and thus were not at a detectable level that would be deemed infectious. Likewise, OTUs with a species classification of *Batrachochytrium dendrobatidis* were detected in five samples, but at 9–19 sequence reads per sample.

Of the eight fungal OTUs that are found at significantly higher proportions in toad samples (Fig 4, Table 2), most were previously sequenced Ascomycota or Basidiomycota yeasts commonly found in plants, soils, or waterways, particularly in colder or alpine/subalpine habitats from other reported datasets [55,56], and including some culturable representatives. For example, in other reports, *Phenoliferia* was also recovered from alpine soils in Colorado [55], and separately in Austrian alpine soils (unpublished UNITE entry). Sequences from *Dactylaria* were also identified in two studies of the Colorado alpine region in soil [55,57]. An OTU classified as *Phaeosphaeria* was also reported in irrigation water from the pond in Lithuania [58] and soil in Alaska (unpublished UNITE entry). Sequences from *Dactylaria* and Volucrispora were also identified in the same irrigation water study as *Phaeosphaeria* [58]. *Phaeosphaeria* contains some plant pathogens and parasites, but the large genetic variation in this group makes it difficult to infer what function they might play in the toad skin community. The *Cladosporium* OTU, which also matches its teleomorph taxonomy *Mycosphaerella* in UNITE, has also been reported from *Salsola soda* leaves in France (unpublished UNITE entries). This group is found ubiquitously, in indoor and outdoor air, human and animal lungs, in the seawater, as plant pathogens, and mycoparasites [59–62]. Additionally, strains of unidentified *Cladosporium* have been isolated from infections on amphibian skin, resulting from injury on the skin being contaminated from the environment, causing skin lesions and spreading to internal organs [53,54]. Many of the putatively host-associated fungal taxa we identified are not found in other animal host systems in the UNITE database, but we expect this is because fungi are understudied in amphibians and the strains likely to be on our toads are more likely to overlap with strains found in the same environment than on other distantly-related amphibia. The fungal taxa identified as putatively host-associated on CO boreal toads also include varying numbers of culturable members, and have been cultured off of boreal toads recently (unpublished data).

## Discussion

Using ITS fungal sequences, we were able to discover that fungal communities differ between the different life stages of the host and its habitat, but that the host and habitat are fairly similar in their fungal communities. As an exception, we identified eight fungal OTUs associated with eggs compared to sediment or water habitats.

A main question in many host systems, including boreal toad skin, is to what extent host versus habitat factors play a role in microbial community composition and assembly [19,63]. In the context of toads, this question has to also consider the constantly changing physiology and environment throughout metamorphosis and how these are intimately linked. The egg and tadpole stages are in an aquatic environment and the animal slowly transitions to a more terrestrial lifestyle throughout metamorphosis, although continuing to spend some of its time in water. Over the course of a toad’s development, age and available skin surface area (size of the animal) might also affect microbial assembly. Prest et al. proposed that hatching and metamorphosis act as disturbances to the bacterial skin community, where each disturbance provided a blank canvas for which microbes to find niche space, and the more time or space they have to occupy that niche space could determine richness and variation within a community [22]. In addition, immune function and/or skin chemistry could affect microbial community assembly, as these functions are affected by metamorphosis and are hypothesized to play a large role in host-microbe interactions in other amphibian species [64–66].

Habitat (aquatic versus terrestrial lifestyles) and host (age and skin surface area) factors confound each other by the very nature of the toad life cycle, but our data suggest that there is likely linkage between the host mycobiome and the fungi found in its habitat. Most toad samples were very similar compared to the environment (Fig 2C), but tadpoles were strikingly similar in their fungal richness and dispersion to water samples (Fig 2A and 2B). Early-stage tadpoles emerge from the viscous outer egg matrix and then spend most of their time swimming through shallow pond water, which could explain why water and tadpoles harbored similar richness and dispersion in their fungal communities (Fig 2A and 2B). Tadpoles are unique compared to other life stages because they have non-keratinized skin cells, spend most of their time swimming through the water column, and feed on algae and small pond invertebrates. In addition, subadults and adults had much higher richness, lower dispersion, and unique beta diversity compared to other samples (Fig 2, S3 Table), suggesting that metamorphosis and/or the associated change in habitat may stabilize the mycobiome to be less variable and more diverse. This makes sense given Prest et al.’s findings in bacteria in this same set of samples [22].

ANCOM analysis supported this high similarity between host and habitat fungal communities–there were a limited number of fungal OTUs associated with the outside of eggs, and no other explicitly toad-associated fungi. While it is possible that tadpoles pick up some of the microbes on the outside of the eggs when they hatch, no tadpole-associated fungal taxa were identified. This tight connection between host and habitat is suggested also in Fig 2C and in past studies of amphibian environments [63,67]. This dynamic makes it difficult to identify host-association, especially through sequence data. Even so, indicator species analyses like ANCOM can successfully leverage large microbial community datasets with host and habitat samples to determine whether certain microbial taxa are likely to interact with hosts, as seen in ruminants, fish, humans, coral, and amphibians [35,67–71]. Once identified, host-associated indicator taxa can be further targeted in culture-based experiments or used in microbial community manipulation studies, if deemed potentially relevant to host biology [37–39]. Most such studies focus exclusively on bacteria, yet ectotherms tend to form strong connections with many microbial taxa in their habitats, notably fungal pathogens [14]. Indeed, one of the fungal OTUs associated with eggs, *Cladosporium*, is part of a group known to contain pathogens that infect eggs and other life stages, and cause a disease called chromomycosis, where a fungus essentially overtakes and consumes the animal’s tissues [53,54,72]. Many of these pathogens are found worldwide and are usually soil and plant saprophytes in the habitat when not found in this specific association with a host [53,54,72]. Fungal pathogens can also form competitive and mutualistic interactions with other fungi in the mycobiome, which in turn affect host health [5–7].

Most of the fungal taxa we identified in the results section were not pathogens, but were previously found or isolated from plants, water, or soil in alpine habitats (e.g., *Phenoliferia*, *Dactylaria*, and *Phaeosphaeria*) [55,56,73,74]. In addition, ectotherms’ body temperatures are strongly influenced by the habitat temperature, hence their microbiomes may be selected to be able to rapidly adapt to seasonal and climate-scale temperature changes [14–19,75–77]. A past study also found that some amphibians seem to select for low abundance habitat bacteria on their skin [26]. This supports the idea that toad skin can be seeded by microbes through their intimate interactions with their habitat (i.e., burrowing, swimming). Many of the fungal taxa we identified have not been found in association with a vertebrate host in the past, but we believe this is due to a lack of host-associated fungal sequence data, particularly from amphibian hosts, and not because there is no association. In addition, most of the eight egg-associated fungal taxa have many culturable representatives, which makes future culture-based studies to confirm host-association possible. While this study examines the fungal taxa on amphibians during the summer season, it is likely that these communities change seasonally and across individuals, however, we were unable to study this variation.

Ecological information on the fungal taxa we identified as egg-associated gives us some insight into alternative hypotheses for fungal host-association in boreal toads. It is possible that fungi from the habitat are simply inoculated onto the host’s skin from the environment, but they could also be transferred by parents onto eggs (vertical transmission), as in other amphibians [78–80]. Boreal toads, however, exhibit less parental care than the species in these cited studies. Fig 2D and S4 Table support that eggs are distinct in their fungal communities from adults (PERMANOVA, R^2^ = 0.1091, p = 0.008). A bacterial taxa study of the same samples presented here found that many sediment and water bacteria were also found on eggs [22], but that there was little overlap with adult or other life stages’ bacterial taxa, indicating that the habitat is a more likely source for boreal toad egg bacteria [22]. In accordance with this finding and previous work that low abundance habitat bacteria can be enriched on some amphibian hosts, we found that although eggs were similar in overall fungal diversity to sediment and water, they had a few key fungal OTUs at higher abundance (Fig 2C, Table 2). In addition, the fungal taxa we identified are mostly habitat-associated in past studies (i.e., plant, soil, water). This further supports the broad pattern we see in the fungal community on the toad host being very similar to the habitat, and including very few biologically relevant host-associated OTUs.

## Conclusion

Despite researchers’ ability to detect many microbes on host animals or plants with newer sequencing techniques, we have far to go in understanding the real nature of host-microbe interactions. In some hosts, like the bobtails squid, termites, or sharpshooters, symbioses are easy to detect and gather evidence for because the symbiont is heavily enriched or the host shows drastically lower survivorship without its symbiont [2,81–84]. Some symbioses may not be so straightforward, and therefore are more difficult to identify, because symbiosis can (1) be on a spectrum between strongly and weakly host-associated [34,85,86], and (2) involve a set of microbial community members, not just a single taxon, that affect host health [8,71,87–90]. It is therefore more complicated to identify symbiotic microbes in systems where the host and habitat are very similar or interlinked, and it is also possible that symbioses with fungi are rare in such hosts.

Microbial amplicon sequencing data has enabled a broader scope of studies of various types of microbial communities that have not been studied in depth in the past. Sequencing datasets can be large in scope and require an approach that reduces the candidate taxa for further study, such as previous work that has aimed to identify beneficial probiotic candidates [37–39,88,91]. In diverse host-associated microbial systems, it can be difficult to identify the type of association between microbes detected via amplicon sequencing and the host. In other studies of ectotherm bacterial microbes, researchers have suggested that because they share a temperature close to that of the environment, it is hypothesized that their skin microbial communities may be more similar and it is harder to determine a “host” community. While the temperature of ectotherms is shared with their environment, the chemistry of the skin environment is unique and likely influences the ability of various microbes to establish [14,15,17,19,67]. Past work has also found that a select few, rare habitat microbes are enriched for on some amphibians [26]. Consistent with this idea, we found significant differences across life stages and similarity between toad versus habitat samples, with many fungal taxa shared among host and habitat. Out of thousands of fungal OTUs, only eight were identified as putatively host-associated, on only one life stage (eggs). Post-metamorphic life stages seemed to be more stable (less variability in their beta diversity) than pre-metamorphic stages, suggesting that this host change has some effect on the fungal community, as has been observed in bacteria in the past [22]. Meanwhile, host-associated taxa may still be specifically tied to host health, for example, in cases of dysbiosis, interactions with disease pathogens, adapting to anthropogenic change, and co-evolution with symbionts [37–39,87,88,92–95]. Sample collection from animals and their environments for microbial sequencing is relatively simple, compared with culturing and isolating microbes, and allows for a larger dataset of potential host-associated microbes to evaluate. This predictive framework could be applied to many host-associated datasets, especially those based on amplicon sequences, and provides a straightforward method for targeted culturing to test symbiosis-related hypotheses [37–39,88,92–94]. Recognition of the microbes associated with wildlife and their importance to host health is growing, and it is changing the way we view the role of microbial communities in animal conservation [37–39,88,92–95].

## Supporting information

S1 Fig Number of samples that dropped out of the analysis and those that ended up in the final dataset used for ecological analyses. Samples dropped out either because PCR could not amplify any genes, because they were filtered out for having low reads or bad quality reads after sequencing, or because certain sample types (indicated here with colors) were not useful for answering our specific hypotheses. Of note, tadpoles were especially difficult to amplify ITS DNA from, but still were highly represented in our final dataset.
(TIF)

S2 Fig Three different alpha diversity measures: Fungal OTU richness, Simpson’s diversity metric, and Shannon diversity index. The respective Kruskal-Wallis chi-squared statistics and Bonferroni-corrected p-values are listed at the top of each graph. The different sample types are listed in the x-axis, with younger developmental stages followed by the specific Gosner stage (i.e., Tadpole.20.22 includes tadpoles that were identified at the Gosner stages 20–22).
(TIF)

S3 Fig The cumulative probability distribution of the W stat. The ANCOM model chooses the W stat cutoff at the highest unchanging (plateaued) cumulative probability. Here we show the cumulative probability distribution of the W stat of the ANCOM comparisons of (A) sediment-eggs and (B) water-eggs, which were the only two pairwise comparisons to yield any significant results.
(TIF)

S4 Fig Rarefaction curve showing the number of OTUs versus the number of sequences subsampled from each sample type. Sample types are indicated by color.
(TIF)

S5 Fig A boxplot of the dispersion of fungal microbial communities across life stages, with high resolution of tadpole stages in particular. This is an alternative version of Fig 2B from the main text. Since the various early and late tadpoles have an even distribution across the whole range of tadpole sample dispersion, we concluded that the tadpole dispersions were not significantly different and therefore could be combined into one sample type for the duration of the analyses in the paper.
(TIF)

S1 Table A table of the 22 OTUs identified by Decontam as being contaminants in this dataset. Taxonomy was determined via the UNITE database version 7.2 2017-12-01 on USEARCH version v10.0.240_i86linux64. Most of these OTUs were rare in our dataset and only one negative control had more than 8 sequences.
(XLSX)

S2 Table European Nucleotide Archive (ENA) submission accession numbers. We submitted our sequences to the European Nucleotide Archive for access by other researchers, under project accession PRJEB41738 (ERP125566). This submission encompasses the extended dataset, including samples that were not used in the analyses reported in this paper.
(XLSX)

S3 Table PERMDISP p-value results. Each sample type was tested in a pairwise fashion and each p-value and confidence interval was calculated using the Tukey’s “Honest Significant Difference” method. Asterisks indicate p-values deemed significant (p ≤ 0.05).
(XLSX)

S4 Table Pairwise PERMANOVA among pairs of sample types, with R^2^ values and FDR-corrected p-values for each pairing. Only significant sample types are listed (FDR-corrected p-value < = 0.005). Numbers after younger developmental stages are the specific Gosner stage. This table lists only significantly different pairwise comparisons. Since none of the separate tadpole stages were significantly different and some had low sample size, all tadpole samples were pooled for hypothesis testing.
(XLSX)
