# Supplementary figures and images for: Identifying fungal-host associations in an amphibian host system
Source: PLoS One. 2021 Aug 19;16(8):e0256328. doi: 10.1371/journal.pone.0256328 (PMC8376043; doi:10.1371/journal.pone.0256328)

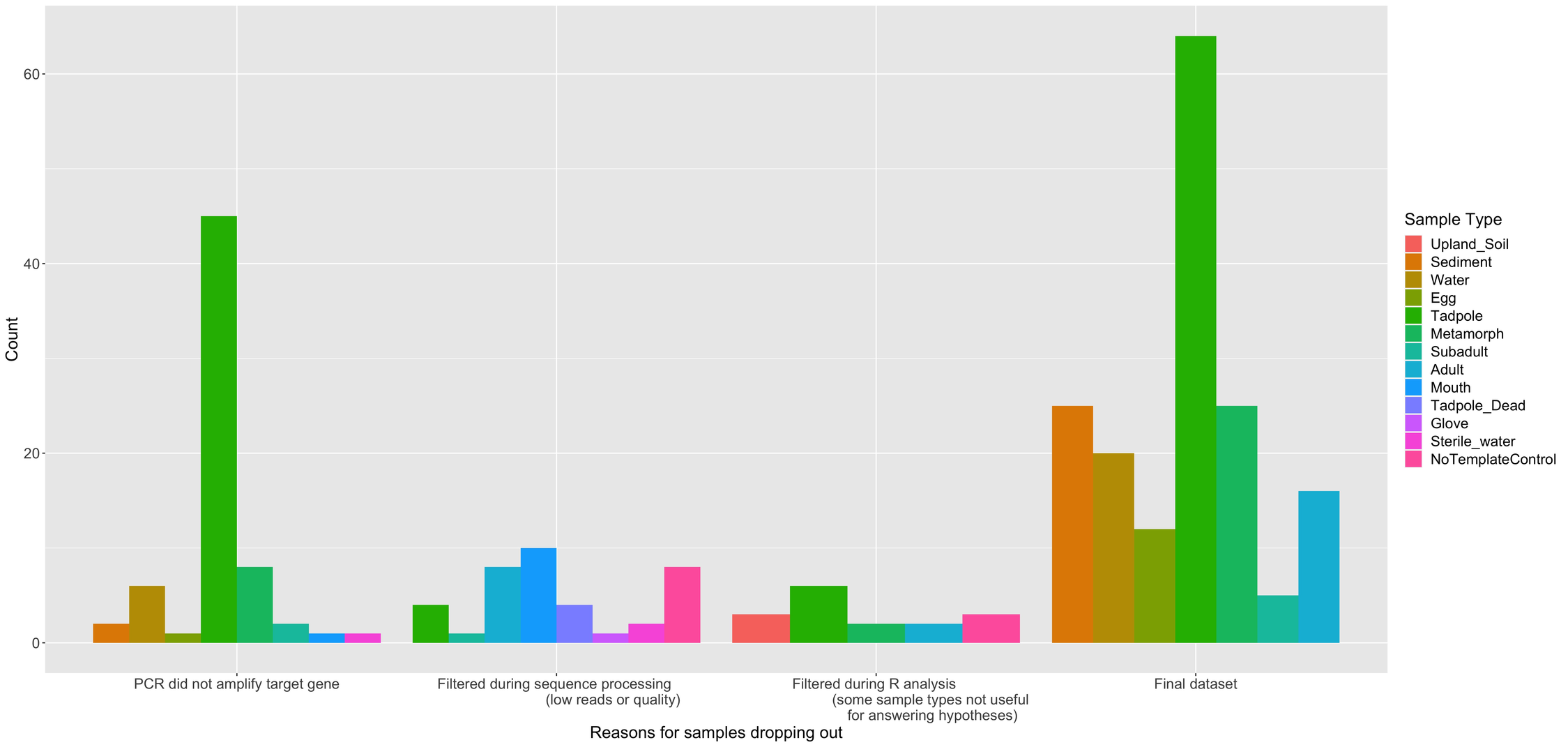

Supplement: S1 Fig — Samples dropped out either because PCR could not amplify any genes, because they were filtered out for having low reads or bad quality reads after sequencing, or because certain sample types (indicated here with colors) were not useful for answering our specific hypotheses. Of note, tadpoles were especially difficult to amplify ITS DNA from, but still were highly represented in our final dataset. (TIF) [file pone.0256328.s001.tif]

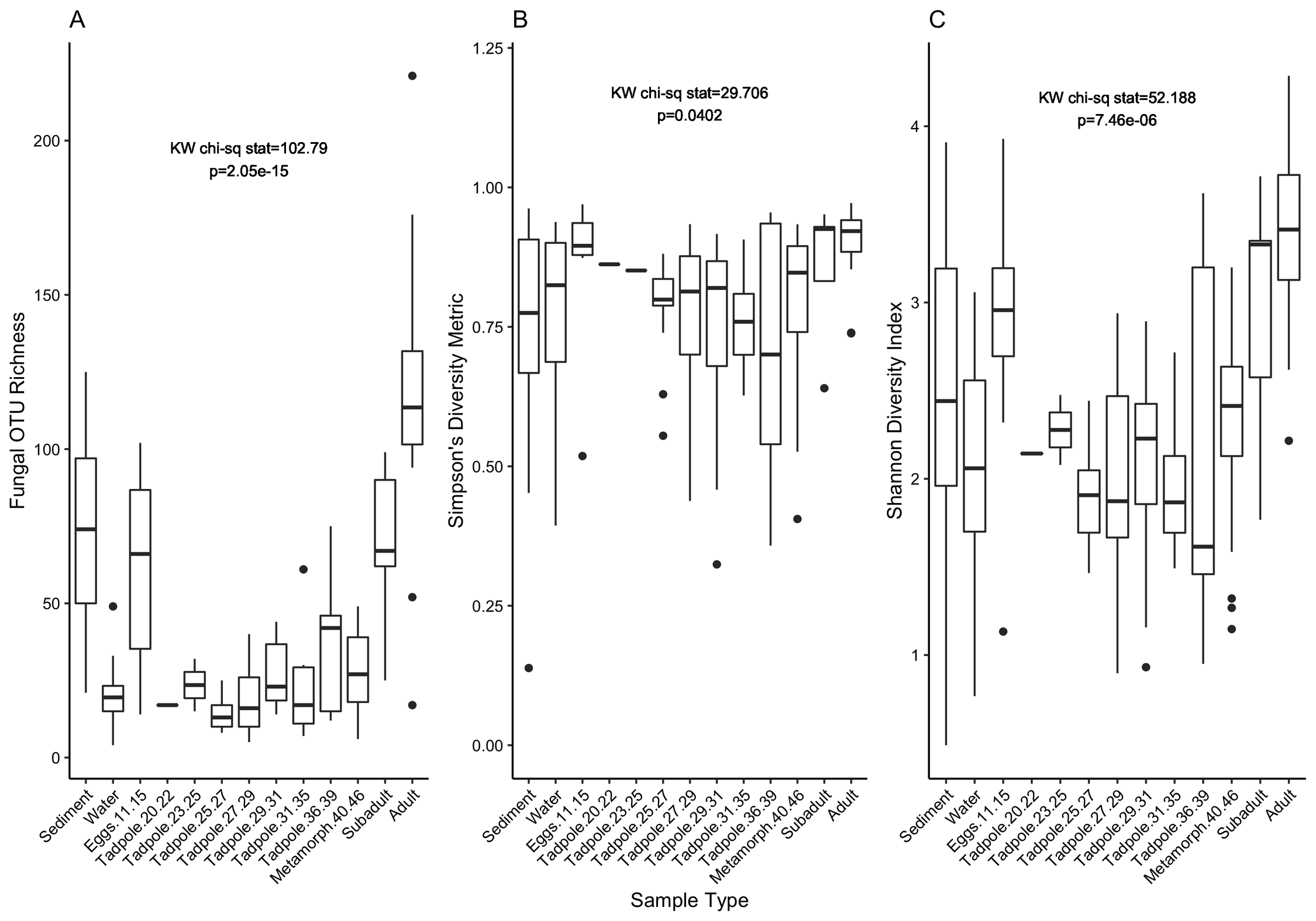

Supplement: S2 Fig — The respective Kruskal-Wallis chi-squared statistics and Bonferroni-corrected p-values are listed at the top of each graph. The different sample types are listed in the x-axis, with younger developmental stages followed by the specific Gosner stage (i.e., Tadpole.20.22 includes tadpoles that were identified at the Gosner stages 20–22). (TIF) [file pone.0256328.s002.tif]

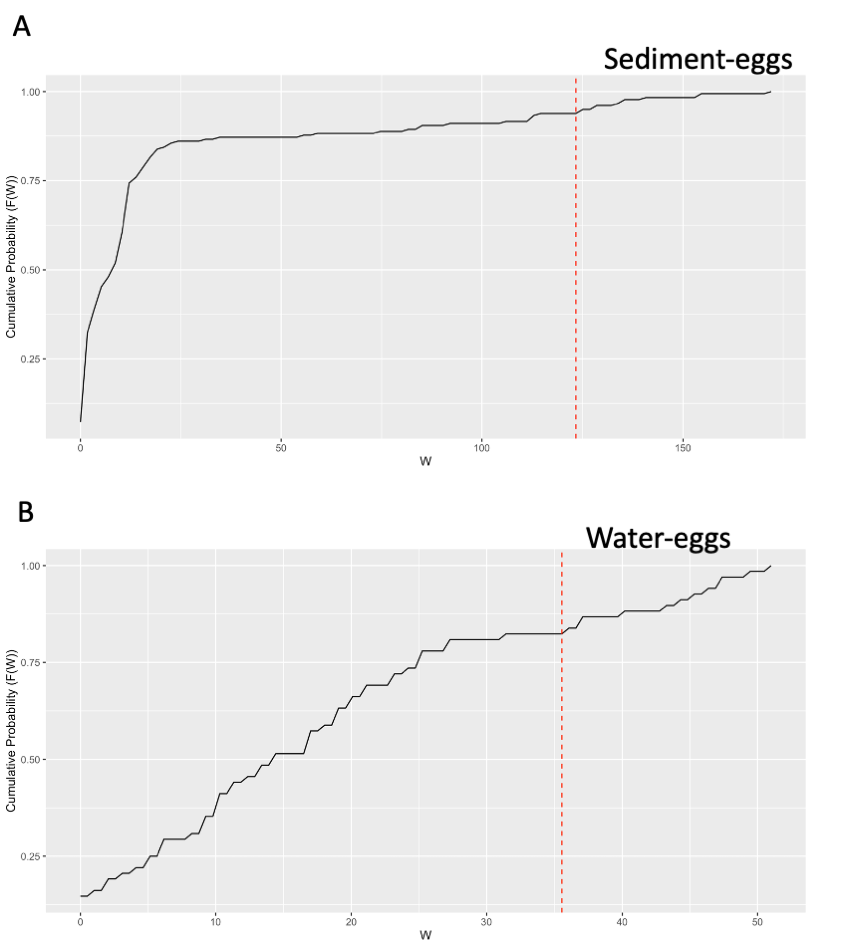

Supplement: S3 Fig — The ANCOM model chooses the W stat cutoff at the highest unchanging (plateaued) cumulative probability. Here we show the cumulative probability distribution of the W stat of the ANCOM comparisons of (A) sediment-eggs and (B) water-eggs, which were the only two pairwise comparisons to yield any significant results. (TIF) [file pone.0256328.s003.tif]

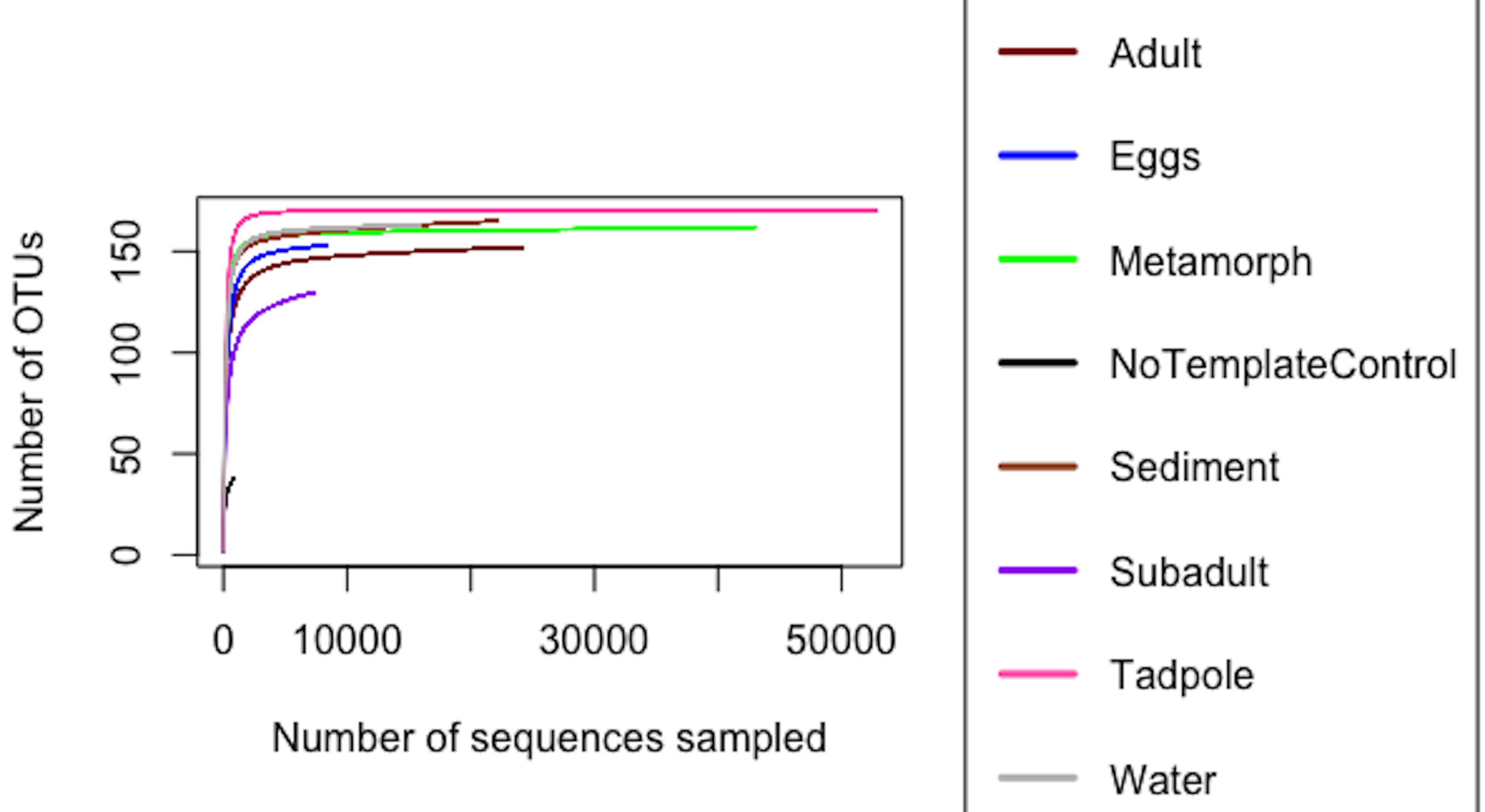

Supplement: S4 Fig — Sample types are indicated by color. (TIF) [file pone.0256328.s004.tif]

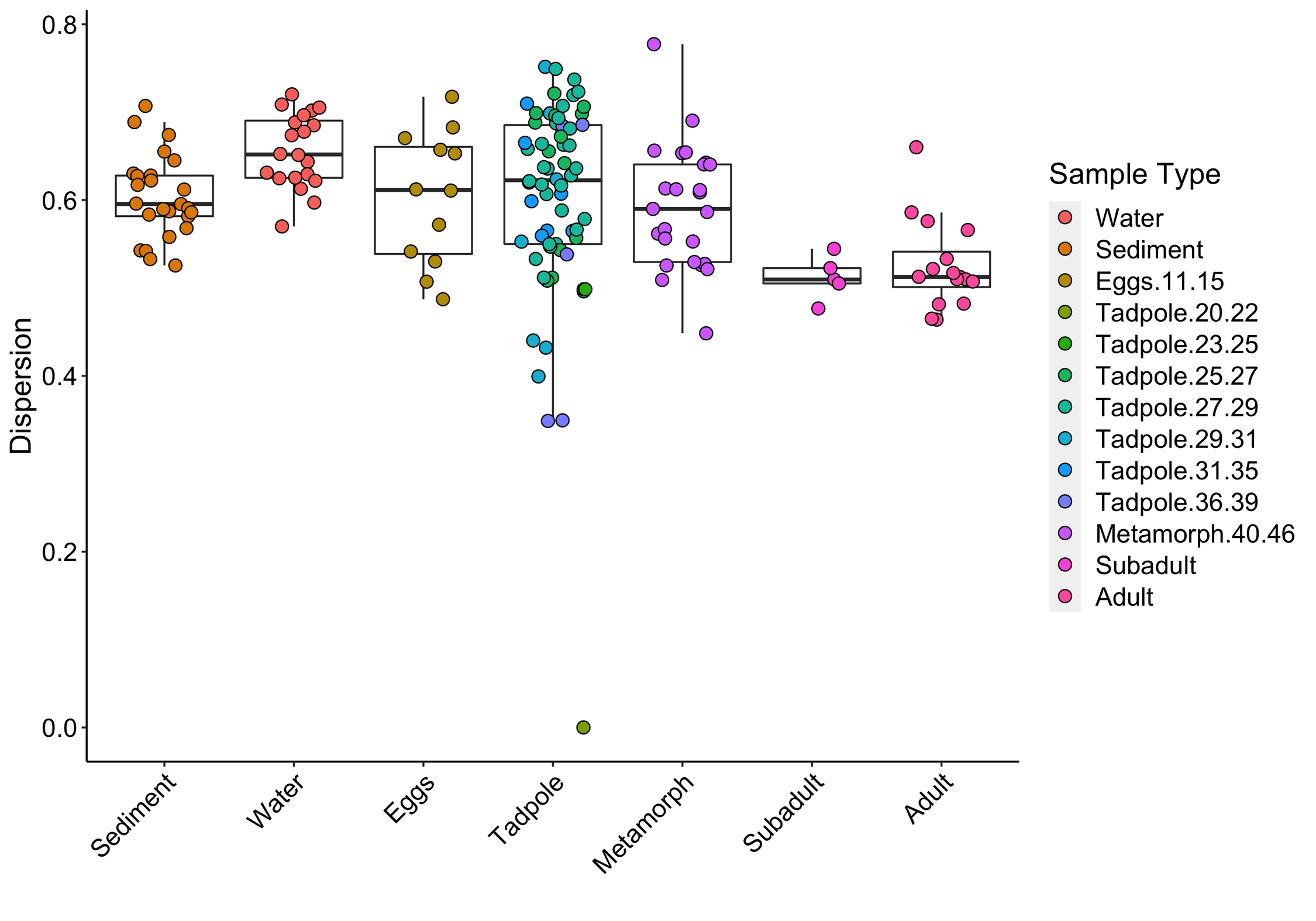

Supplement: S5 Fig — This is an alternative version of Fig 2B from the main text. Since the various early and late tadpoles have an even distribution across the whole range of tadpole sample dispersion, we concluded that the tadpole dispersions were not significantly different and therefore could be combined into one sample type for the duration of the analyses in the paper. (TIF) [file pone.0256328.s005.tif]
